# Adaptive latitudinal variation in Common Blackbird *Turdus merula* nest characteristics

**DOI:** 10.1002/ece3.952

**Published:** 2014-02-21

**Authors:** Mark C Mainwaring, D Charles Deeming, Chris I Jones, Ian R Hartley

**Affiliations:** 1 Department of Biological Sciences, Macquarie UniversitySydney, NSW, 2109, Australia; 2 Department of Biological Sciences, University of LincolnRiseholme Park, Lincoln, LN2 2LG, UK; 3 Lancaster Environment Centre, Lancaster UniversityLancaster, LA1 4YQ, UK

**Keywords:** Insulation quality, latitude, nest composition, nest size, spring temperature, *Turdus merula*

## Abstract

Nest construction is taxonomically widespread, yet our understanding of adaptive intraspecific variation in nest design remains poor. Nest characteristics are expected to vary adaptively in response to predictable variation in spring temperatures over large spatial scales, yet such variation in nest design remains largely overlooked, particularly amongst open-cup-nesting birds. Here, we systematically examined the effects of latitudinal variation in spring temperatures and precipitation on the morphology, volume, composition, and insulatory properties of open-cup-nesting Common Blackbirds’ *Turdus merula* nests to test the hypothesis that birds living in cooler environments at more northerly latitudes would build better insulated nests than conspecifics living in warmer environments at more southerly latitudes. As spring temperatures increased with decreasing latitude, the external diameter of nests decreased. However, as nest wall thickness also decreased, there was no variation in the diameter of the internal nest cups. Only the mass of dry grasses within nests decreased with warmer temperatures at lower latitudes. The insulatory properties of nests declined with warmer temperatures at lower latitudes and nests containing greater amounts of dry grasses had higher insulatory properties. The insulatory properties of nests decreased with warmer temperatures at lower latitudes, via changes in morphology (wall thickness) and composition (dry grasses). Meanwhile, spring precipitation did not vary with latitude, and none of the nest characteristics varied with spring precipitation. This suggests that Common Blackbirds nesting at higher latitudes were building nests with thicker walls in order to counteract the cooler temperatures. We have provided evidence that the nest construction behavior of open-cup-nesting birds systematically varies in response to large-scale spatial variation in spring temperatures.

## Introduction

Animals across a wide range of taxa construct nests in order to create a safe and environmentally suitable location in which to lay eggs and/or raise offspring (Collias and Collias 1984; Hansell 2005). The microclimate within nests directly influences the conditions experienced by the developing offspring, and experimental studies have shown that offspring raised in suboptimal nest microclimates suffer negatively in terms of their growth and development (Peréz et al. 2008; Ardia et al. 2010). As parents are in control of their nest construction behavior, and the design of completed nests influences the internal microclimate (Ar and Sidis 2002; Edelman 2011), then we should expect natural selection to have acted upon parents so that they build an optimal structure for the local environmental conditions. Furthermore, there may be trade-offs in nest design that differ between sites, for example, a larger nest may be better insulated against temperature loss, but may be more easily detected by potential predators (Møller 1990).

Adaptive variation in nest design in relation to variation in environmental conditions remains generally overlooked (Collias and Collias 1984), and to our knowledge, only one study has examined whether nest characteristics vary adaptively over small spatial scales. In that study, patterns of nest site selection and nest design by common amakihi's (*Hemignathus virens virens*) were examined in relation to decreasing ambient temperatures with increasing altitude on Hawaii (Kern and van Riper 1984). Nests were compared at two sites at different altitudes, and those nests built at higher altitudes were placed closer to canopy edges in order to receive more direct sunlight than nests at the site at lower altitude. Further, nests at higher altitudes also had denser, but not thicker, walls that contained greater amounts of cup lining material and had greater insulation capacities than nests built at the site at a lower altitude (Kern and van Riper 1984). This study suggests that birds are able to gauge variation in environmental conditions over small spatial scales and adjust their nest-building behaviors to create a suitable microclimate for the offspring and attending parent/s (Hansell 2000, 2005). Consequently, nest characteristics should also be expected to vary adaptively over large spatial scales, such as with increasing ambient temperatures as latitude decreases.

Intraspecific studies in birds have shown that American Robin *Turdus migratorius* and Yellow Warbler *Dendroica petechia* nests were heavier and had thicker nest walls in northern Canada than in southern Canada (Rohwer and Law 2010; Crossman et al. 2011). Meanwhile, the amount of cup lining material and insulatory properties of both Blue Tit *Cyanistes caeruleus* and Great Tit *Parus major* nests decreased with decreasing latitude and increasing spring temperatures in Great Britain (Mainwaring et al. 2012), and the insulatory properties of northern oriole *Icterus spp* nests decreased as latitude decreased in North America (Schaefer 1980). Consequently, nest characteristics appear to vary adaptively over large spatial scales, yet our understanding is ecologically and taxonomically limited to a few studies that have focused disproportionately on burrow-nesting rodents and hole-nesting birds. Both groups nest in concealed locations and suffer comparatively low levels of nest predation (Martin 1993; Martin and Briskie 2009), meaning that they may have more scope than open-nesting species to adjust the design of their nests to create suitable nest microclimates. This is important as minimizing the risk of predation is an important challenge for nesting birds (Lima 2009), and natural selection favors the construction of small nests, and particularly so amongst open-cup-nesting species (Martin 1993; Antonov 2004). Therefore, birds at northerly latitudes may face a trade-off between the need to build a well-insulated nest with the need to build one small enough to minimize the risk of predation. Consequently, our understanding of adaptive variation in nest design over large spatial scales would benefit from studies of open-nesting species (Hansell 2000). Further, previous studies of open-cup-nesting birds (Rohwer and Law 2010; Crossman et al. 2011; but see Schaefer 1980) have examined nests from two study sites, thereby leaving open the possibility that site-specific effects such as predation risk and habitat quality influenced the females’ nest-building behaviors or the design of the completed nests.

**Figure 1:**
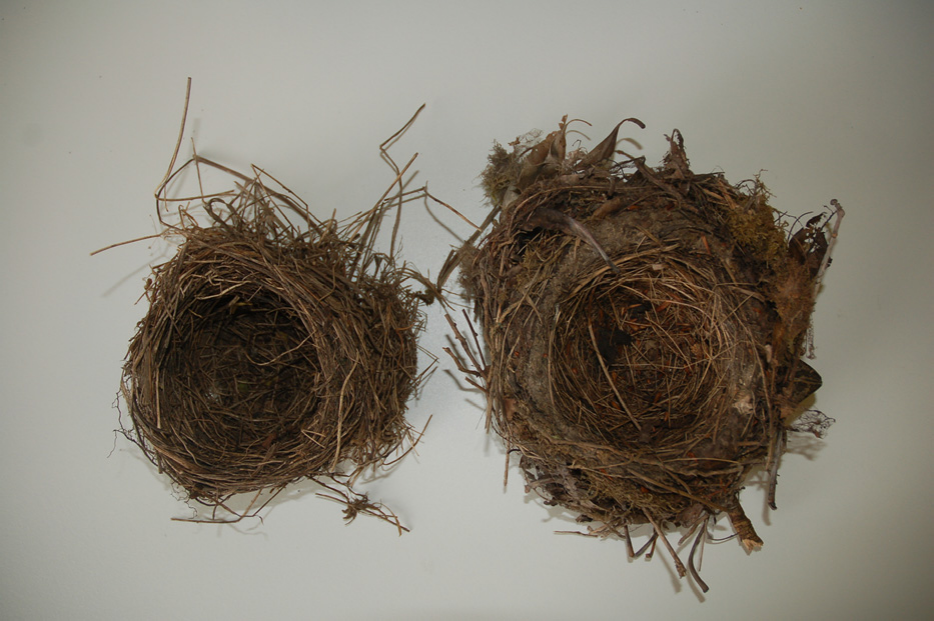
Common Blackbirds’ (*Turdus merula*) nests built at different latitudes. The nest on the right is from Aberdeen and the nest on the left is from Penryn.

In this study, we systematically examined the effects of latitudinal variation in spring temperatures and precipitation on the volume, morphology, composition, and insulatory properties of Common Blackbird *Turdus merula* nests (Fig. 1). We chose Common Blackbirds as a study species as they are widespread and numerous throughout Great Britain, and their nests are relatively easy to find. Common Blackbirds are territorially breeding passerine birds that build open-cup nests, and in Great Britain, they are multi-brooded, and the breeding season begins in late February when the female selects the nest site and builds the nest alone (Snow 1958a,b). Common Blackbird nests consist of a bulky cup of twigs, dry grasses, moss, stalks, and other vegetative material plastered on the inside with mud or muddy leaves and completed by the addition of a substantial cup lined with fine grass, thin dead stems, or rootlets (Snow 1958a; Cramp 1988). Females then lay a clutch of 3–5 eggs and incubate the clutch alone, before both parents provision the nestlings. Common Blackbirds have 2–3 broods per year, and so shortly after the nestlings fledge, or when the nest contents are predated, females lay another clutch of eggs either in a freshly built nest or more rarely in the same nest, and the breeding season finishes in late June (Snow 1958a,b1958b; Cramp 1988). In this study, we test three predictions for Common Blackbird nests. First, that as spring temperatures increase with decreasing latitude, the size and morphology of nests will decrease as the need for insulation declines. Second, that the mass of the various building materials will exhibit an inverse relationship with spring temperature. Third, that nest insulatory properties will decrease as the need for insulation diminishes with warmer spring temperatures.

## Methods

### Study areas and general methods

**Figure 2:**
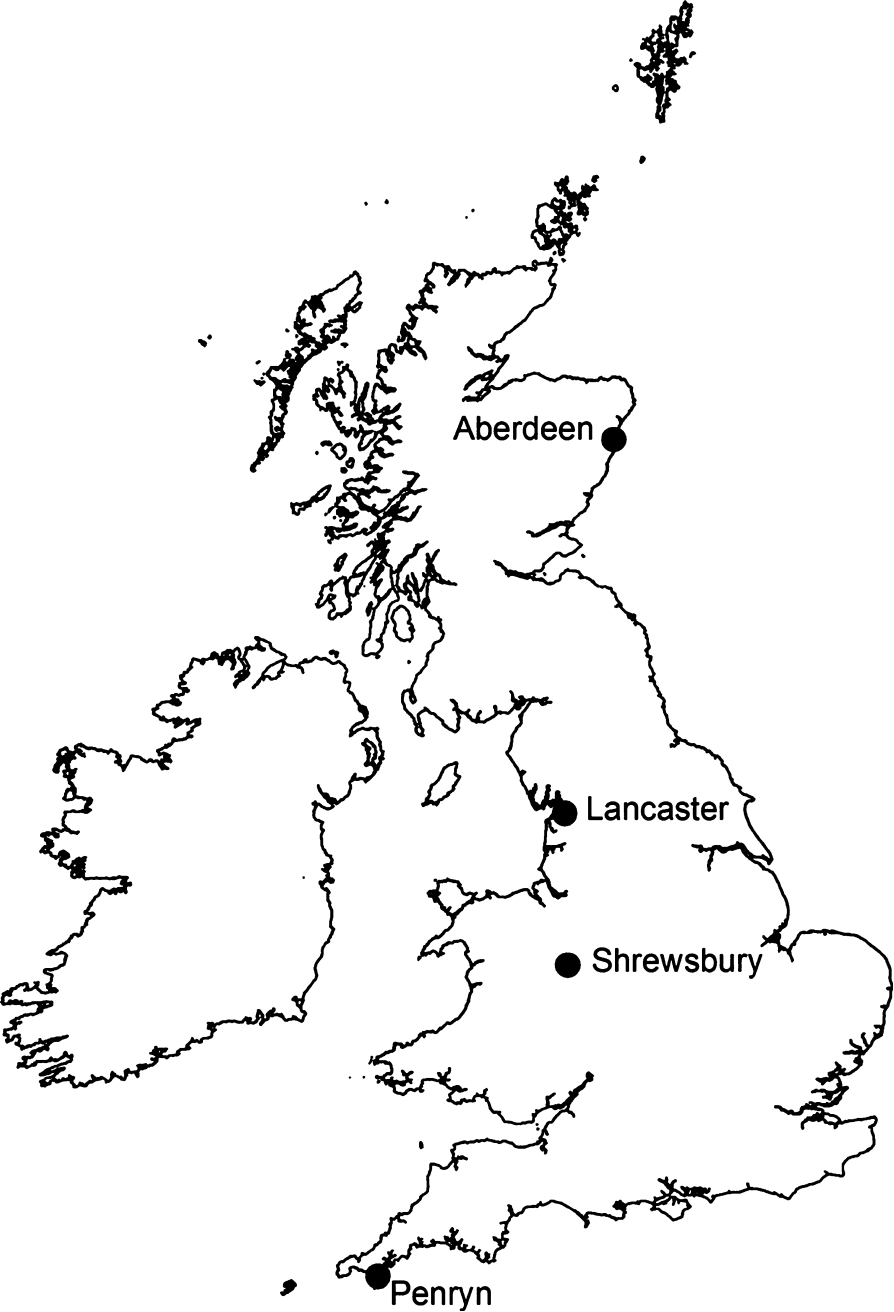
The location of the four study sites within Great Britain from which Common Blackbirds’ nests were collected in order to examine the effects of latitudinal variation in spring temperatures on their size, morphology, composition, and insulatory properties.

Data were collected from nests constructed in March–June 2012 at four study sites; Aberdeen (57.10°N; *n* = 14), Lancaster (54.00°N; *n* = 14), Shrewsbury (52.43°N; *n* = 14), and Penryn (50.05°N; *n* = 15); spread over 7° of latitude in Great Britain (Fig. 2). At each site, suitable areas of habitat were searched regularly for Common Blackbird's nests, but once located the nests were left alone until the end of July when they were collected and placed into sealed and labeled plastic bags, before being transported to Lancaster University.

### Quantifying nest insulatory properties

All nests were frozen at −20°C for at least 4 days in order to kill any invertebrates present within the nests, before the insulatory properties of all of the nests from each of the study sites were determined. Nests were placed in a controlled condition environment of 15°C and 70% humidity for a minimum of 72 h (following McGowan et al. 2004; Mainwaring et al. 2012) before the insulatory properties were quantified in a 10°C constant temperature and 70% constant humidity room. Nests were placed onto a hard surface, and two iButtons (Maxim: DS1922L) were heated to 80°C with the “nest” iButton gently pushed into the base of the nest cup and the “control” iButton placed 5 cm away on an adjacent hard surface and at the same height. Then, both the “nest” and the “control” iButtons were left to cool for 30 min, while they recorded the temperature every 20 sec. The cooling rates of the nests were used to quantify the insulatory properties of the nests (following McGowan et al. 2004; Mainwaring et al. 2012).

Cooling rates were obtained for each nest by fitting the empirical temperature data to logistic models of the form: temperature of iButton – ambient temperature + *b*e^−*ct*^, where *b* is the initial temperature gradient between the iButton and ambient, *c* describes the cooling rate of the iButton, *t* is the time since the experiment began in seconds, and e is the base of natural logarithms. The nest insulatory properties were calculated as the differences in *c* of the “nest” and “control” iButtons, with a large positive difference indicating high nest insulation (following McGowan et al. 2004; Mainwaring et al. 2012). The insulatory properties of all nests were quantified three times, with the iButtons placed in the same place each time, and the mean of the three scores was used in the analyses.

### Quantifying nest volume, morphology, and composition

The volume and morphology of nests were quantified in two ways. First, we measured five aspects of nest morphology: external nest diameter (±0.05 cm), internal nest-cup diameter (±0.05 cm), external nest depth (±0.05 cm), internal nest-cup depth (±0.05 cm), and nest wall thickness (±0.05 cm). The external nest diameter is the mean of the maximum and minimum diameters of the outer nest cup, and the internal nest-cup diameter is the mean of the maximum and minimum diameters of the inner nest cup. The external nest depth was quantified as the distance from the top rim of the nest walls to the bottom of the nest exterior. The internal nest-cup depth is the distance from the top of the rim of the nest walls to the base of the interior nest cup, where the eggs would be. Meanwhile, the nest wall thickness was quantified as the mean of eight evenly spaced measurements of the nest wall. Then, as the nests of open-cup-nesting birds such as Common Blackbirds are approximately shaped as half a spheroid, the volume of nests was calculated by determining the outer nest volume from the following equation: nest volume = 2/3*πa*^2^*b*, where “*a*” is the largest, and “*b*” is the smallest value of the outer radius and the height of the nest, respectively (following Møller 1990).

Then, the nests were left to dry for 1 week before their composition was quantified by dissecting them into their component materials: twigs, dry grasses, moss, other vegetative material, mud, muddy leaves, thin dead stems, rootlets, and miscellaneous material (Snow 1958a,b1958b; Cramp 1988). Miscellaneous material compromised various nonvegetative materials such as small pieces of polythene and plastic, nylon chord, fishing line, and magnetic tape. Components were weighed on an electronic balance to the nearest 0.05 g. All measurements were taken by one observer (CIJ) meaning that there was no error due to interobserver variability.

### Spring temperature and precipitation data

Spring temperature and precipitation data were obtained from the British Atmospheric Data Centre. We used the “Met Office – MIDAS Land Surface Stations data (1853–current)” data set to calculate the mean average of the maximum and minimum daily temperatures and the mean average amount of precipitation, from the nearest available (10.75 ± 5.17 km) meteorological stations to each of the study sites. Common Blackbirds at lower latitudes breed earlier than Common Blackbirds at higher latitudes (Snow 1958a; Cramp 1988), and so data were obtained for 8 week/56 day periods beginning on April 1, 7, 14, and 21 for the Penryn, Shrewsbury, Lancaster, and Aberdeen study sites, respectively.

### Statistical analyses

Data were analyzed using the SPSS version 20.0 (Armonk, NY) statistical package. We tested for variation in the volume, morphology, composition, and insulatory properties of nests in relation to latitudinal variation in spring temperatures and precipitation using linear mixed models. We used mixed models as they allowed us to control for the possible nonindependence of nests collected within the same study sites by including “study site” as a random effect in the analyses (Crawley 1993). First, separate models had the various nest size measurements (“external nest diameter,” “internal nest-cup diameter,” “external nest depth,” “internal nest-cup depth,” “nest wall thickness,” and “nest volume”), the various nest materials (“total nest,” “twigs,” “dry grasses,” “moss,” “vegetative material,” “mud,” “muddy leaves,” “thin dead stems,” “rootlets,” and “miscellaneous material”), and “nest insulatory property” as dependent variables, and all models had “spring temperature” included as a fixed explanatory covariate term and “study site” as a random term. We then repeated these analyses with “precipitation” included as the fixed explanatory covariate term, before further partitioning the variance of significant models using one-way analysis of variance (ANOVA) and *post-hoc* Tukey tests.

We then explored the roles of nest morphology, volume, and composition in explaining variation in nest insulatory properties. We began by calculating the repeatability (*r*) values for the cooling rates used to quantify the nest insulatory properties over each of the three tests following the approach suggested by Lessells and Boag (1987). The ANOVA-based approach is a widely used method to calculate repeatabilities and uses the *F* table of an ANOVA with the individual identities treated as factorial predictors. We interpreted the *r*-values following the suggestion of Martin and Bateson (1986), with higher *r*-values indicating higher repeatability scores. Variation in nest insulatory properties was then examined using two linear mixed models. The first model had “nest insulatory property” as the dependent variable, the various nest, and cup dimensions, and their two-way interaction terms as fixed explanatory covariate terms and “study site” as a random term. The second model had “nest insulatory property” as the dependent variable, and the various nest materials and their two-way interaction terms as fixed explanatory covariate terms and “study site” as a random term. Both of the maximal models included all of the explanatory variables, and their two-way interaction terms, and explanatory variables were then assessed for significance when they were the last terms in the model. Nonsignificant effects were then sequentially removed by stepwise deletion until the most parsimonious models were obtained, and individual factors are also presented if they were present in a significant interaction term (Crawley 1993). All statistical tests were two-tailed, means are presented ±1 SE, and a critical *P*-value of 0.05 was applied throughout.

## Results

**Figure 3:**
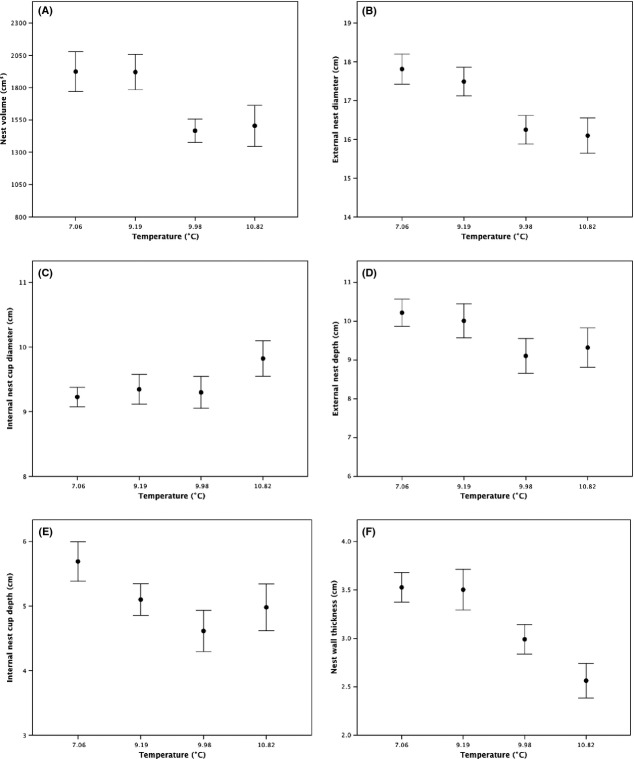
The relationship between latitudinal variation in spring temperatures and (A) nest volume, (B) external nest diameter, (C) internal nest-cup diameter, (D) external nest depth, (E) internal nest-cup depth, and (F) nest wall thickness of Common Blackbirds’ nests from four sites in Great Britain. Data are shown as mean ± SE.

**Figure 4:**
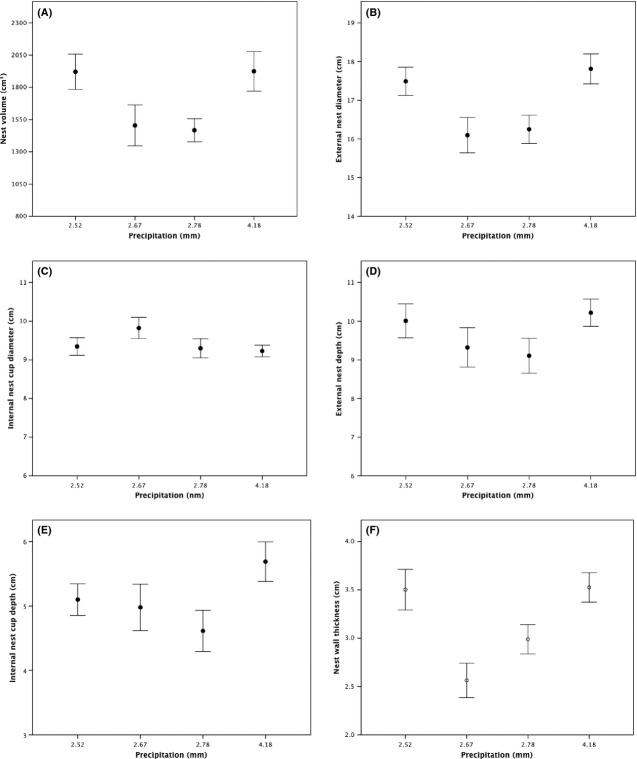
The relationship between latitudinal variation in precipitation and (A) nest volume, (B) external nest diameter, (C) internal nest-cup diameter, (D) external nest depth, (E) internal nest-cup depth, and (F) nest wall thickness of Common Blackbirds’ nests from four sites throughout Great Britain. Data are shown as mean ± SE.

The mean spring temperatures at the study sites increased as latitude decreased (Aberdeen = 6.81 ± 0.29°C, Lancaster = 9.18 ± 0.31°C, Shrewsbury = 10.30 ± 0.34°C, Penryn = 11.36 ± 0.30°C: *F*_3,220_ = 10.82, *P* < 0.001), but there was no latitudinal variation in the mean levels of precipitation (Aberdeen = 4.18 ± 0.36 mm, Lancaster = 2.52 ± 0.19 mm, Shrewsbury = 2.78 ± 0.12 mm, Penryn = 2.67 ± 0.09 mm: *F*_3,220_ = 0.04, *P* = 0.872). We explored the effects of latitudinal variation in spring temperatures and precipitation on the volume, morphology, composition, and insulatory properties of the nests using mixed models and *post-hoc* Tukey tests. The volume of nests did not vary with either spring temperatures (*F*_3,57_ = 3.71, *P* = 0.197; Fig. 3A) or precipitation (*F*_3,57_ = 0.571, *P* = 0.529; Fig. 4A), but there was variation in terms of the morphology of nests. The external diameter of nests showed no variation with precipitation (*F*_3,57_ = 1.14, *P* = 0.398, Fig. 4B), but declined with increasing temperatures (*F*_3,57_ = 21.54, *P* = 0.019, Fig. 3B), with *post-hoc* tests revealing that nests from Penryn had smaller diameters than nests from Aberdeen (*P* = 0.018) but other differences were nonsignificant (all *P* values >0.05). However, the internal diameter of nests showed no significant variation with increasing temperatures (*F*_3,57_ = 2.36, *P* = 0.130, Fig. 3C) or with precipitation (*F*_3,57_ = 4.03, *P* = 0.060, Fig. 4C). The thickness of the nest walls showed no relationship with precipitation (*F*_3,57_ = 0.58, *P* = 0.524, Fig. 4F), but declined with increasing temperatures (*F*_3,57_ = 15.08, *P* = 0.027, Fig. 3F), with *post-hoc* tests indicating that nest walls were thicker in Penryn than in Shrewsbury (*P* = 0.001) and that nests from Shrewsbury had thicker walls than nests from Lancaster and Aberdeen (*P* = 0.002), which did not differ (*P* = 0.318). Meanwhile, the external nest-cup depth did not vary with latitudinal variation in temperatures (*F*_3,57_ = 3.13, *P* = 0.083, Fig. 3D) nor precipitation (*F*_3,57_ = 1.09, *P* = 0.407, Fig. 4D). There was a marginally significant trend toward internal nest-cup depths being shallower as temperatures increased with decreasing latitude (*F*_3,57_ = 4.32, *P* = 0.042, Fig. 3E), with *post-hoc* tests revealing no significant differences between any of the individual study sites (all *P* values >0.05). Meanwhile, internal nest-cup depths did not vary with latitudinal variation in precipitation (*F*_3,57_ = 3.02, *P* = 0.060, Fig. 4E).

**Figure 5:**
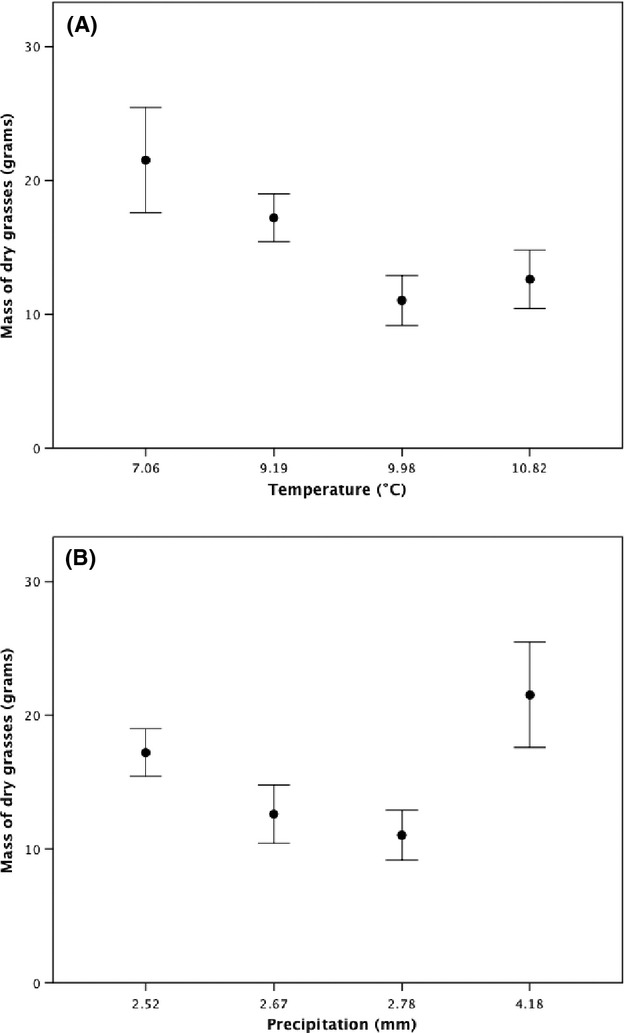
The relationship between latitudinal variation in (A) spring temperatures and (B) precipitation, and the mass of dry grasses within Common Blackbirds’ nests from four study sites throughout Great Britain. Data are shown as mean ± SE.

The quantity of dry grasses within nests decreased with increasing spring temperatures at lower latitudes (*F*_3,57_ = 8.70, *P* = 0.005, Table 1, Fig. 5A), with *post-hoc* tests revealing that nests from Penryn had smaller diameters than nests from Aberdeen (*P* = 0.018) but other differences were nonsignificant (all *P* values >0.05). However, there was no relationship with precipitation (*F*_3,57_ = 2.61, *P* = 0.248, Table 1, Fig. 5B). There was also a marginally significant trend toward the quantity of mosses declining with increasing spring temperatures at lower latitudes (*F*_3,57_ = 4.04, *P* = 0.049, Table 1), with *post-hoc* tests revealing no significant differences between any of the individual study sites (all *P*-values >0.05), but there was no variation with precipitation (*F*_3,57_ = 0.427, *P* = 0.580, Table 1). Meanwhile, there was no latitudinal variation in any other of the composite materials used to construct the nests (all *P* values >0.05, Table 1).

**Table 1:** Latitudinal variation in the composition of Common Blackbirds’ nests at four study sites throughout Great Britain. Note that miscellaneous materials are defined in the methods. Values are means ± SE.

Study site	Total nest mass (grams)	Mass of twigs (grams)	Mass of dry grasses (grams)	Mass of moss (grams)	Mass of vegetative material (grams)	Mass of mud (grams)	Mass of muddy leaves (grams)	Mass of thin dead stems (grams)	Mass of rootlets (grams)	Mass of miscellaneous (grams)
Aberdeen	213.50 ± 28.60	7.73 ± 3.31	21.54 ± 3.93	8.39 ± 1.96	9.82 ± 0.95	143.43 ± 23.80	13.15 ± 3.20	5.55 ± 1.28	1.49 ± 0.41	0.45 ± 0.17
Lancaster	208.27 ± 25.47	4.95 ± 0.91	17.22 ± 1.79	8.80 ± 2.54	16.47 ± 7.20	133.78 ± 22.30	16.03 ± 4.13	4.83 ± 1.11	3.69 ± 1.59	0.47 ± 0.27
Shrewsbury	184.71 ± 25.32	6.88 ± 1.96	11.05 ± 1.88	4.54 ± 1.35	8.16 ± 0.71	129.69 ± 20.74	13.30 ± 3.25	5.57 ± 0.86	3.29 ± 1.77	0.77 ± 0.28
Penryn	176.84 ± 15.28	3.76 ± 0.99	12.62 ± 2.18	3.42 ± 1.84	8.38 ± 0.70	130.66 ± 14.41	9.35 ± 1.96	3.84 ± 0.84	2.12 ± 0.56	1.27 ± 0.44

**Figure 6:**
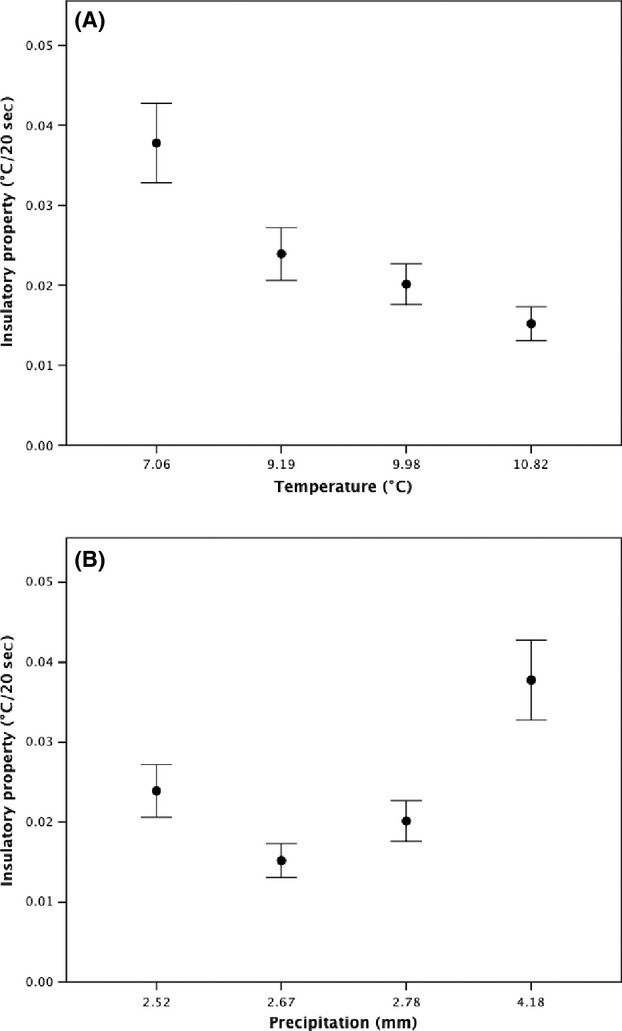
The relationship between latitudinal variation in (A) spring temperatures and (B) precipitation, and the insulatory properties of Common Blackbirds' nests from four study sites throughout Great Britain. Data are shown as mean ± SE.

**Figure 7:**
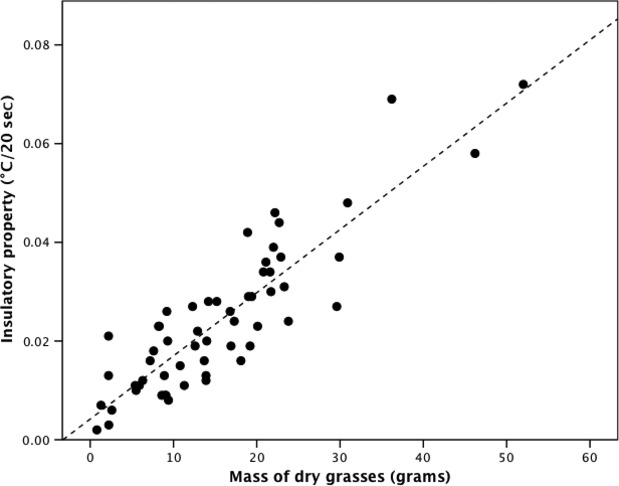
The relationship between the mass of dry grasses and the insulatory properties of Common Blackbirds’ nests from four sites throughout Great Britain.

Nest insulatory properties decreased significantly with increasing ambient temperatures as latitude decreased (Aberdeen = 0.38 ± 0.005°C/20 sec, Lancaster = 0.24 ± 0.003°C/20 sec, Shrewsbury = 0.20 ± 0.003°C/20 sec, Penryn = 0.15 ± 0.002°C/20 sec: *F*_3,57_ = 25.40, *P* < 0.001, Fig. 6A), but there was no relationship with latitudinal variation in the amount of precipitation (*F*_3,57_ = 7.96, *P* = 0.102, Fig. 6B). Reassuringly, the repeatability of the nest insulatory property scores across the three tests was significant and high for both iButtons (nest iButton: *r* = 0.80, *F*_1,57_ = 18.35, *P* < 0.001; control iButton: *r* = 0.83, *F*_1,57_ = 15.82, *P* < 0.001). The insulatory property of individual nests was positively correlated with the mass of dry grasses within nests (*F*_3,57_ = 17.36, *P* = 0.001, Fig. 7), but not with any other of the volume or morphology measurements taken or the mass of any of the composite materials (all tests, *P* > 0.05).

## Discussion

We have provided evidence that the nest construction behavior of an open-cup-nesting bird species varies adaptively in a systematic manner in response to large-scale spatial variation in spring temperatures, but not precipitation. Importantly, by examining nests from four study sites along a latitudinal gradient, we have significantly reduced the chances that site-specific effects such as breeding habitat quality, predation pressure, and nutrient availability may have influenced the females’ nest-building behaviors or the design of the completed nests (Crossman et al. 2011; Mainwaring et al. 2012). Unfortunately, we do not know the precise dates on which the nests were built nor the subsequent dates on which eggs were laid, which means that we are unable to examine possible seasonal changes in the volume, morphology, composition, and insulatory properties of the nests (Mainwaring and Hartley 2008; Deeming et al. 2012). Further, we cannot exclude the possibility that some nests were used as breeding sites twice during the spring, although the reuse of nests is rare (Snow 1958a,b; Cramp 1988). Nevertheless, we remain confident that latitudinal variation in such nest leftacteristics represents biologically meaningful patterns.

The insulatory properties of the Common Blackbird's nests decreased with warmer temperatures at lower latitudes, thereby supporting our third prediction, which posited that nest insulatory properties would decrease as the need for insulation diminished with warmer spring temperatures. This suggests that the decreasing spring temperatures were counteracted by the increasing nest wall thickness and by increasing the amount of grass within nest walls. This should help create an optimal microclimate for incubating eggs and brooding nestlings along the latitudinal gradient. The systematic variation in nest morphology and composition to create suitable nest microclimates along the latitudinal gradient also supports our first two predictions, which posited that morphology of nests would decrease as the need for insulation declines in warmer environments and that the mass of the various building materials will exhibit an inverse relationship with spring temperature, respectively. This is important as previous experimental studies have shown that nestlings raised in suboptimal nest microclimates suffer reduced growth and development (Peréz et al. 2008; Ardia et al. 2010; DuRant et al. 2013). Meanwhile, the amount of precipitation at the four study sites did not vary with latitude, and none of the nest leftacteristics varied with the amount of spring precipitation. The absence of any consistent trends with precipitation suggests that spring temperatures are a much better predictor of nest leftacteristics. This may be expected as incubating parents protect eggs from precipitation, meaning that precipitation may not be expected to determine nest design (Kern and van Riper 1984; Heenan and Seymour 2011). However, it is prudent to consider that the microenvironment in which individual nests were located may have also influenced the design of nests, and while the Common Blackbirds in our study may have positioned nests in dry conditions, such as directly under logs and branches, it may be more difficult to ameliorate the effects of ambient temperatures (Crossman et al. 2011). Further studies could usefully examine the relative influence of a range of environmental variables, such as temperature, precipitation, wind speed, and cloud cover, on nest-building behaviors and nest design.

The amount of grass incorporated into nests increased in cooler environments at higher latitudes and the insulatory property of individual nests, irrespective of study site, was positively correlated with the amount of grass within nests. While these two lines of evidence suggest that incorporating grass increased the insulatory quality of nests, a previous study showed that of a range of commonly used nesting materials, grass was the material that provided the worst insulation (Hilton et al. 2004). This apparent disparity may be explained by the fact that in our study, grass was incorporated into nests with thicker nest walls, meaning that it was the thickness of the nest walls generally, and not the amount of grassy material in the nests *per se* that resulted in those nests having greater insulation properties. Irrespective of the exact function of the various nesting materials, however, the construction of thermally optimal nests in cooler environments must be traded off against both the associated energetic costs of constructing a larger nest (Skowron and Kern 1980; Mainwaring and Hartley 2009, 2013) and the requirement to build smaller nests that minimize the risk of predation from visually searching predators.

Avoiding predation is a continuous challenge for nesting birds (Lima 2009), and natural selection exerts strong selection pressures on the design of nests, particularly amongst open-cup-nesting birds (Martin 1993; Martin and Briskie 2009). Empirical studies have shown that larger nests of open-cup-nesting species are predated more frequently than smaller nests (Antonov 2004; Lima 2009). In Common Blackbirds, observational studies have shown that nests with greater external diameters (Gregoire et al. 2003) and nests that are more detectable (Cresswell 1997) suffer disproportionately high levels of predation, and an experimental study showed that artificially enlarged nests were predated more frequently than nests that remained unchanged in size and nests that were made artificially smaller (Møller 1990). Reassuringly, previous reports of nest dimensions for this species (Bocheński 1968; Pikula 1983) are comparable to data presented here, suggesting that the nests in our study are typical for Common Blackbirds. Nevertheless, the selection for nests with smaller diameters could also be inferred from our study, as while the internal nest-cup diameters of nests did not vary along the latitudinal gradient, nests in cooler environments at higher latitudes had thicker walls and larger external diameters. This suggests that those Common Blackbirds living at higher latitudes were forced to build wider nests than their conspecifics living in warmer environments. This may simply keep the nest microclimate within acceptable limits for embryonic development (Webb 1987) or may reflect a response of the adult to the prevailing temperature, which is unrelated to the requirements of the eggs or chicks and more related to the thermal needs of the incubating female (Britt and Deeming 2011). It does suggest that those Common Blackbirds living in warmer environments at more southerly latitudes were building nests that were wide enough to provide structural support for developing offspring and attending parent (Collias and Collias 1984; Heenan and Seymour 2011), but not any larger as the relatively benign environmental conditions enabled them not to. Therefore, female Common Blackbirds in the northerly study areas may have been trading off the need to build a well-insulated nest with one that was small enough to minimize the risk of predation. Consequently, the possibility of a trade-off between building a large and well-insulated nest with one that is small enough to minimize the risk of predation is clearly a fruitful area for further research.

The marginally significant trend toward a decline in the amount of mosses incorporated into nests as spring temperatures increased suggests that moss may have a insulatory role or is important in bulking out the nest wall. While it is unlikely that any of the materials used in Common Blackbird nests will be limited at any particular location, the potential for changing environmental conditions to impact upon the availability of materials for nest building remains unclear. For example, mud is an important component of nests of many other species (Hansell 2000), and it has been suggested that it may become limited as increasing temperatures cause soils to become drier and harder to penetrate by birds (Tomialojc 1992). Further, it was postulated that the limited availability of mud may have been an important factor in restricting the nesting distribution of the Song Thrush *Turdus philomelos* (Tomialojc 1992). Meanwhile, increasing ambient temperatures are predicted to increase the availability of nutrients in the soil, which is likely to decrease the distribution and growth of mosses through increased competition from vascular plants (O'Neil 2000; Jägerbrand et al. 2003). While this study suggests that Common Blackbirds are currently able to collect sufficient amounts of mud to build their nests, further temporal changes in environmental conditions as a result of anthropogenic climate change may mean that materials such as mosses may become a limited resource in the future (Mainwaring et al. 2012). Further research could therefore usefully examine the effects of rising temperatures on the availability of nesting materials for birds.

Our study suggests that open-cup-nesting birds systematically vary the design of their nests in response to large-scale latitudinal variation in spring temperatures, but not precipitation. Accordingly, there are three areas of research that are likely to prove fruitful in the future. First, further research should aim to examine the generality of our findings by examining spatial variation in nest leftacteristics over larger continental scales both in similar open-cup-nesting species and in ecologically different hole-nesting species, as the two are likely to construct nests while under different selection pressures. Such studies should aim to examine larger numbers of nests collected from more sites across multiple years. Second, it would be interesting to quantify the availability of various nesting materials, such as grasses, mosses, and mud in order to examine whether the availability of nesting materials differs between study sites. Third, it would be useful to examine how the thermal properties of nests influence the length of incubation periods and the phenotypic quality of neonates under natural incubation conditions.
